# Decoding Solubility Signatures from Amyloid Monomer Energy Landscapes

**DOI:** 10.1021/acs.jctc.4c01623

**Published:** 2025-02-24

**Authors:** Patryk Adam Wesołowski, Bojun Yang, Anthony J. Davolio, Esmae J. Woods, Philipp Pracht, Krzysztof K. Bojarski, Krzysztof Wierbiłowicz, Mike C. Payne, David J. Wales

**Affiliations:** † Yusuf Hamied Department of Chemistry, University of Cambridge, Lensfield Road, Cambridge CB2 1EW, U.K.; ‡ Shenzhen College of International Education, Antuoshan sixth Road, Shenzhen 518040, China; § Theory of Condensed Matter Group, Cavendish Laboratory, Department of Physics, University of Cambridge, Cambridge CB3 0HE, U.K.; ∥ Department of Engineering, University of Cambridge, Trumpington Street, Cambridge CB2 1PZ, U.K.; ⊥ Department of Physical Chemistry, Gdansk University of Technology, Narutowicza 11/12, Gdansk 80-233, Poland; # Department of Biochemistry and Molecular Genetics, University of Virginia School of Medicine, 1335 Lee Street, Charlottesville, Virginia 22908, United States

## Abstract

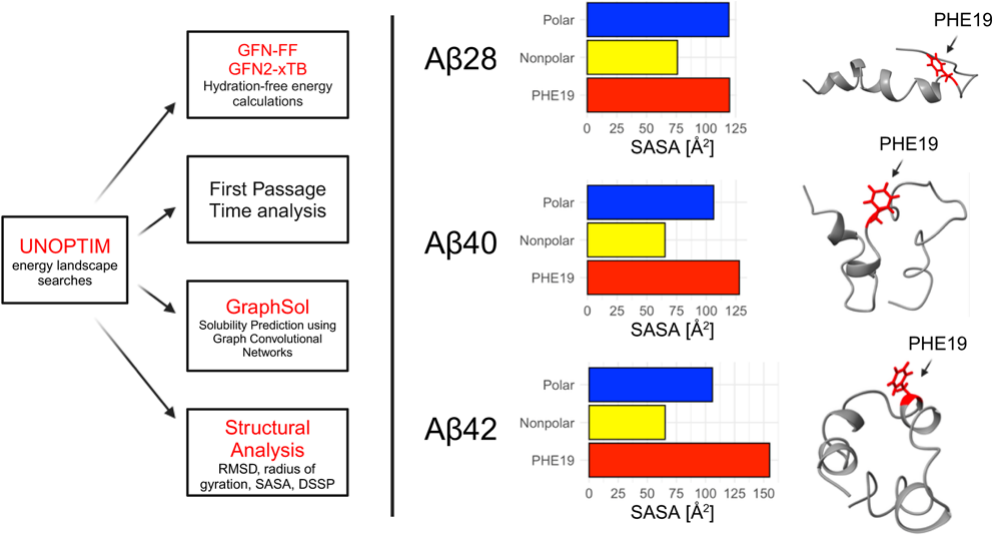

This study investigates the energy landscapes of amyloid monomers, which are crucial for understanding protein misfolding mechanisms in Alzheimer’s disease. While proteins possess inherent thermodynamic stability, environmental factors can induce deviations from native folding pathways, leading to misfolding and aggregation, phenomena closely linked to solubility. Using the UNOPTIM program, which integrates the UNRES potential into the Cambridge energy landscape framework, we conducted single-ended transition state searches and employed discrete path sampling to compute kinetic transition networks starting from PDB structures. These kinetic transition networks consist of local energy minima and the transition states that connect them, which quantify the energy landscapes of the amyloid monomers. We defined clusters within each landscape using energy thresholds and selected their lowest-energy structures for the structural analysis. Applying graph convolutional networks, we identified solubility trends and correlated them with structural features. Our findings identify specific minima with low solubility, characteristic of aggregation-prone states, highlighting the key residues that drive reduced solubility. Notably, the exposure of the hydrophobic residue Phe19 to the solvent triggers a structural collapse by disrupting the neighboring helix. Additionally, we investigated selected minima to determine the first passage times between states, thereby elucidating the kinetics of these energy landscapes. This comprehensive approach provides valuable insights into the thermodynamics and kinetics of Aβ monomers. By integration of multiple analytical techniques to explore the energy landscapes, our study investigates structural features associated with reduced solubility. These insights have the potential to inform future therapeutic strategies aimed at addressing protein misfolding and aggregation in neurodegenerative diseases.

## I Introduction

**Figure 1:**
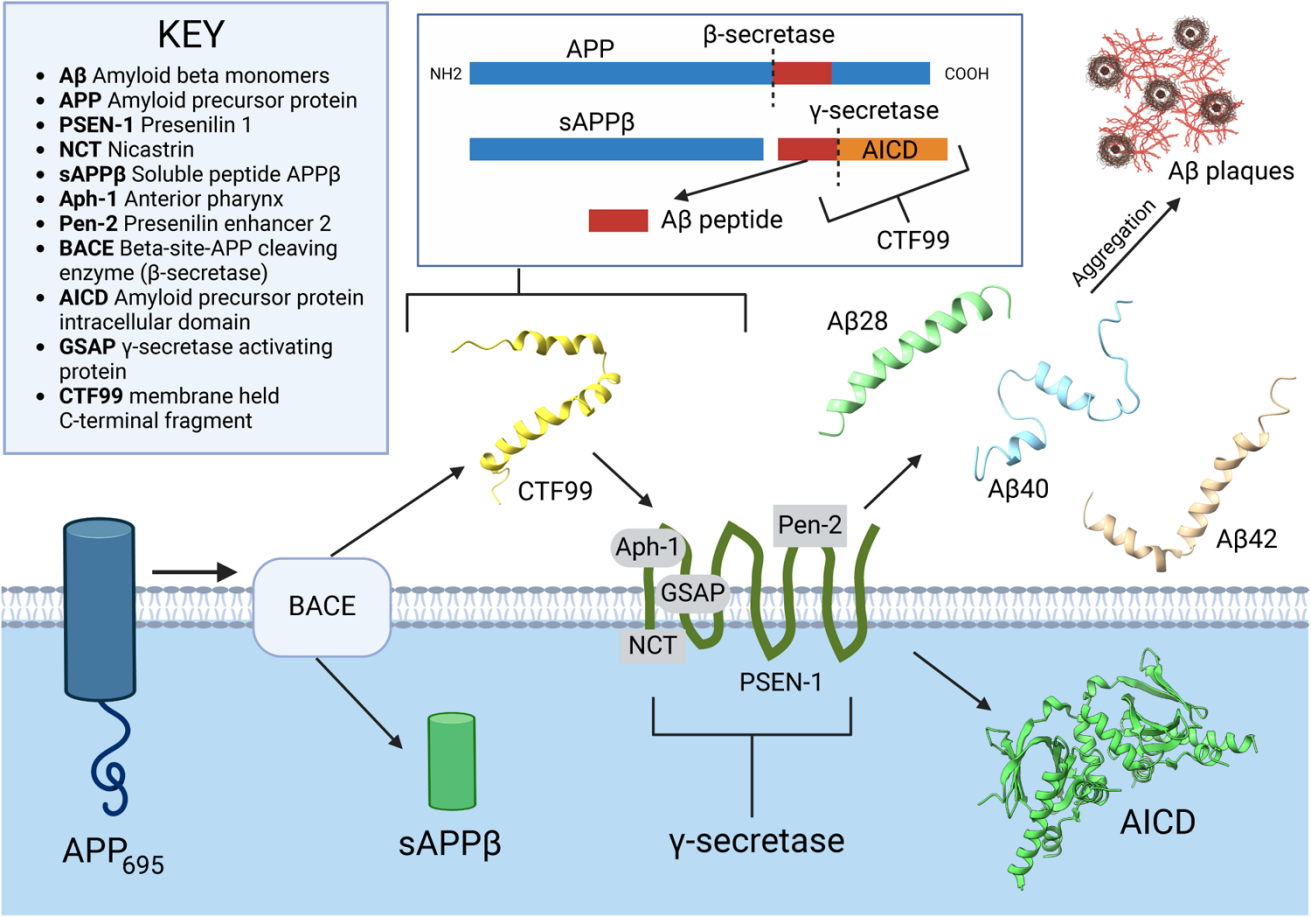
Cleavage of APP695 residue protein first by β-secretase to form CTF-99 (PDB ID: 2LP1), then by γ-secretase subsequently to form Aβ monomers of Aβ40 (PDB ID: 1AML) and Aβ42 (PDB ID: 1IYT) residues. Aβ28 (PDB ID: 1AMC) is a monomer consisting of the same residues as the first 1–28 residues from the full Aβ42 peptide, and it has been extensively explored in previous work.^7^ This sequence is followed by the AICD domain (PDB ID: 3DXC) at the C-terminal end. The figure was created with BioRender.com.

Proteins are among the most abundant biomolecules in the human body and intricately govern a wide range of physiological functions. Their precise folding into a compact conformation, termed the native state, is essential for proper functioning. This folding process, corresponding to a descent down an energy funnel, has been conceptualized by Leopold et al.^1^ The structural transformations that occur during protein folding result in concurrent decreases in the enthalpy and internal entropy. At or below optimal folding temperatures, the enthalpy change (Δ*H*) outweighs the entropy term (*T*Δ*S*) leading to a thermodynamically favorable process characterized by a reduction in Gibbs free energy, resulting in the acquisition of the native state.^1,2^ Despite the inherent thermodynamic stability of protein folding, specific environmental conditions may divert proteins from their native state, causing trapping in local energy minima.^3,4^ These external factors include, among others, temperature, pH, and molecular crowding. Internal factors, such as mutations or chemical modifications including oxidation or glycation, can also affect folding.^5^ These diversions from native folding may involve energy barriers, which impede proteins from regaining their native conformation.^2^ Consequently, misfolding events can lead to the formation of aggregates (Figure 1).^6^

The formation of protein aggregates, commonly referred to as amyloids, is important in various scientific disciplines, including chemistry, biology, and medicine.^8^ Amyloid β (Aβ) monomers are not inherently problematic and may serve functional purposes,^9^ such as protecting mature neurons from excitotoxic death.^10^ However, amyloid formation disorders are a great concern for the aging human population, with Alzheimer’s disease (AD) serving as a prominent example. AD is a progressive brain disorder that affects memory, thinking skills, and the ability to perform daily tasks. The median survival of AD is 5.8 years, with approximately 55 million people affected worldwide in 2019.^11^ AD is characterized by the presence of plaques in the brain primarily composed of Aβ proteins and Tau fibrils, collectively termed “Aβ-plaques” (Figure 1).^12^ Maintaining the delicate balance of protein damage levels is ordinarily regulated by adjusting chaperone and protease levels.^8^ However, when the generation of misfolded proteins surpasses the rate at which they are destroyed or refolded, aggregates can accumulate.^13−15^ In AD, Aβ peptides result from the cleaving of the amyloid precursor protein (APP), a Type I integral membrane glycoprotein composed of 695–770 residues^16^ with diverse metabolic functions.^12^

APP695, the predominantly expressed protein found in the human brain at significantly higher levels,^17,18^ is sequentially cleaved by β-secretase (e.g., BACE1) and γ-secretase (a multiunit integral membrane protease^16^) through proteolytic processing, yielding a range of Aβ proteins that form the key components of amyloid fibrils in AD patients.^19^ Aβ peptides are secreted outside the cell membrane, aggregating to form neurotoxic plaques (Figure 1).^20^ In familial Alzheimer’s disease (FAD), an inherent mutation in APP or presenilin (a component of the γ-secretase complex) causes increased cleavage-producing Aβ monomers.^20^ In sporadic diseases, the buildup of Aβ monomers may be attributed to a decrease in Aβ degrading enzymes.^21^ The primary amino acid sequence of Aβ40 and Aβ42 (Figure 1), elucidated in 1984,^22^ provided insights into the accumulation mechanisms. These proteins, which belong to the class of intrinsically disordered proteins,^23^ undergo various structural transitions, and their aggregation into forms such as oligomers, protofibrils, and amyloid fibrils contributes to the pathology of AD.^12^ Aβ28 is composed of the first 28 residues of the extracellular domain^24^ and lacks the anchoring transmembrane domain compared to the more abundant Aβ40 and Aβ42 monomers^25,26^ and thus exhibits significant structural differences. Its fibrillogenic properties in terms of plaque formation are also explored and compared in this paper as a benchmark system.

After cleavage from APP695, monomeric forms of Aβ40 and Aβ42 have been proposed to adopt unstructured conformations,^27^ which then normally change to an α-helical structure upon binding with a negatively charged biological lipid.^27^ Both the random coil and the α-helical structures are relatively soluble in solution. The multifunnel energy landscape^23,28^ describes multiple competing folding pathways, where Aβ peptides can settle into less soluble, metastable states, often forming partially folded intermediates resembling native-like structures.^29^ Specifically, this process includes a rearrangement of a soluble random coil and α-helical structures into insoluble β-sheet aggregated structures that are fibrillar.^27^ Proteins do not spontaneously transform from a state of lower energy to higher energy. Thus, they aggregate only when the amyloid state has lower free energy, making the process thermodynamically favorable.^30^ For example, when Aβ peptides are present at high concentrations, they become highly unstable thermodynamically, which causes them to undergo aggregation. This process ultimately results in the formation of insoluble plaques.^31^

Investigation into the solubility of Aβ monomers is essential for the diagnosis of patients with Alzheimer’s disease, since both the soluble and insoluble forms of the Aβ peptide affect the brain. For instance, measuring only the concentration of insoluble Aβ peptides cannot distinguish between patients with high pathology control and those with Alzheimer’s.^32^ In contrast, the concentration of soluble Aβ peptides, particularly Aβ40, shows a clear inverse correlation with synapse loss, providing a more effective marker for differentiation.^32^ While soluble Aβ is better at predicting change in synapses of Alzheimer’s patients,^32^ insoluble Aβ forms neurotoxic plaques and aggregates. Amyloid fibrils form because of a decrease in the solubility of the monomer, controlled by supersaturation. Thus, lower solubility and a lower saturation barrier for a particular conformation of these amyloids may imply higher, or earlier, contribution to plaque formation.^33−35^

In a preceding study, we successfully integrated the UNRES (UNited RESidue) coarse-grained potential^36,37^ into the OPTIM^38^ program, resulting in the development of the UNOPTIM program.^39^ In this study, we employ the UNOPTIM^39^ program alongside the discrete path sampling (DPS) approach^40,41^ to construct the kinetic transition network.^42−44^ Through this approach, we analyze the potential energy landscapes of three Aβ monomer proteins: Aβ28 (PDB ID: 1AMC), Aβ40 (PDB ID: 1AML), and Aβ42 (PDB ID: 1IYT). We identified clusters of UNRES energy minima by applying specific energy thresholds, ensuring a comparable number of structures for analysis across all systems. Using specific clustering energy thresholds, the clusters are defined as the sets of local minima that can interconvert without exceeding that threshold value above their lowest member. The lowest-energy structures within these clusters were further examined and are referenced throughout the text as selected minima. Using Graph Convolutional Networks (GCN), we identified solubility trends facilitated by a detailed examination of the energy landscape of amyloid monomers. Additionally, we analyzed transitions between selected minima by employing first passage time (FPT) distributions to investigate the kinetics of these landscapes. By integration of multiple analytical techniques, this study elucidates structural features associated with reduced solubility, potentially contributing to aggregation.

## II Theory and Technical Details

**Figure 2:**
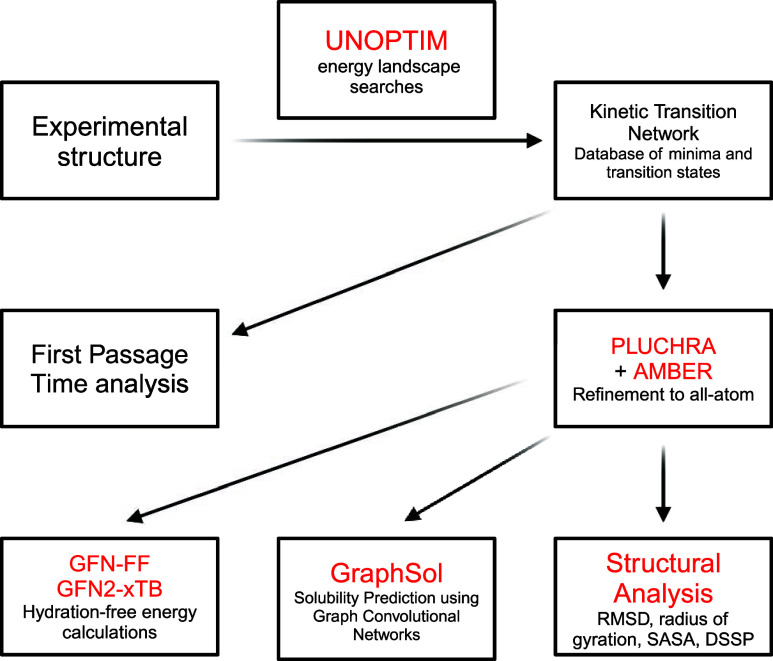
Flowchart illustrating the organization of the calculations used in this work, with the programs highlighted in red. All aspects of the methodology are further detailed in this section.

Here we describe the methodology used in this study. The flowchart in Figure 2 summarizes the flow of data and information between different levels of calculation. All components of the flowchart are further detailed within this section.

### II.A UNOPTIM Program

**Figure 3:**
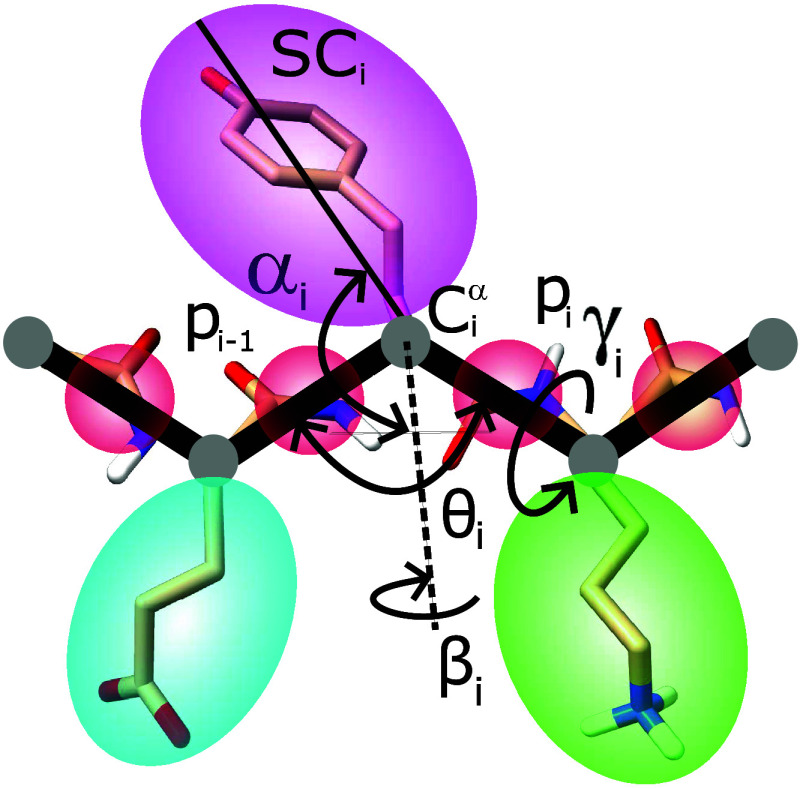
Representation of polypeptide chains with the UNRES model. United side chains are depicted as colored ellipsoids, while united peptide groups appear as red spheres. The specific angles referenced in the image are detailed in the text. The illustration is adapted from ref (51).

The interface between the UNRES^36,37^ coarse-grained potential and the OPTIM program^38^ allows fast calculations with useful accuracy for exploring protein energy landscapes.^39^ The UNRES model was chosen because it remains under constant development, with new extensions added frequently.^45,46^ These refinements help to improve accuracy in simulations of various protein structures with extension to large systems.^47,48^ Furthermore, the structural prediction capabilities of the UNRES model are tested every two years^49,50^ during the Critical Assessment of protein Structure Prediction (CASP) experiments. The model employs two interaction sites per amino acid, which are the united side chains (SCs) and united peptide groups (p), respectively (Figure 3).^36^ The α-carbons (*C*^α^) are not interaction sites but are instead used to define the geometry of the protein main chain. The united peptide groups are found halfway between two consecutive *C*^α^ atoms^51^ (Figure 3). Implicit solvent is used and is computed using interaction potentials involving SCs, coefficients of friction, and stochastic forces.^52^

The UNRES energy function is
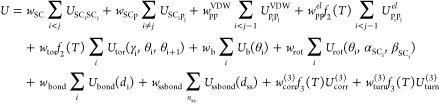
1

The formula is composed of long-range intersite terms (*U*_SC_i_SC_j__, *U*_SC_i_p_j__, *U*_p_i_p_j__), and local terms. Local terms include the virtual-bond-deformation (*U*_bond_), virtual-bond-angle terms (*U*_b_), side-chain rotamer terms (*U*_rot_), and third-order multibody terms (*U*_corr_^(3)^, *U*_turn_^(3)^). These terms account for coupling between backbone-local and backbone-electrostatic interactions.^37^ Virtual-bond vectors are expressed as *C*^α^···*C*^α^ and *C*^α^···*SC*. The polypeptide chain backbone geometry is defined by the virtual-bond angles *C*^α^···*C*^α^···*C*^α^ and virtual-bond-dihedral angles *C*^α^···*C*^α^···*C*^α^···*C*^α^, referred to as θ and γ, respectively. The virtual-bond angle and virtual-bond-dihedral angle for the *i*^th^*C*^α^ (*C*_*i*_^α^) are represented as θ_*i*_ and γ_*i*_. The α_SC_ and β_SC_ angles define the orientation of an SC center with respect to the backbone. Therefore, the *SC*_*i*_ center local geometry can be defined by the spherical angles α_*i*_, referred to as the angle between bisection of θ_*i*_ and the *C*_*i*_^α^···*SC*_*i*_ vector, and the angle of rotation of the *C*_*i*_^α^···*SC*_*i*_ vector from the *C*_*i*–1_^α^···*C*_*i*_^α^···*C*_*i*+1_^α^ plane, described as β_*i*_ (Figure 3).^51^

Each energy term is multiplied by an appropriate weight *w*_*x*_,^53^ and the values corresponding to factors greater than order one are additionally multiplied by temperature coefficients.^54^ These coefficients reflect the influence of the first generalized-cumulant term^55^ on temperature, and are defined by *f*_*n*_(*T*), where *T*_*o*_ = 300 K:^54,56^
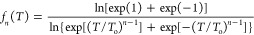
2

### II.B Energy Landscape Exploration and the Discrete Path Sampling Approach

The molecular energy landscape defines thermodynamic and kinetic properties. Here, we employ discrete path sampling (DPS)^40,41^ to explore the landscape using geometry optimization to locate minima of the UNRES energy function and the transition states that connect them.^57^ To initiate DPS, two minima must be first linked by one discrete path, which usually encounters intervening minima.^41^ The set of minima and transition states constitutes a kinetic transition network,^58−60^ which allows us to estimate the thermodynamic and dynamic properties of Aβ monomers.

The OPTIM program uses the Limited-memory Broyden-Fletcher-Goldfarb-Shanno minimization algorithm (LBFGS algorithm)^61^ for minimization. Transition states are refined using hybrid eigenvector-following^62−66^ from candidates found by the doubly nudged^67,68^ elastic band method^69−72^ (DNEB). A transition state is defined as a stationary point where the Hessian matrix has exactly one negative eigenvalue.^73^ To find candidate transition states using the DNEB method, two minima are interpolated by images connected by harmonic springs in an elastic band, and the energy of the bands is minimized by the LBFGS algorithm.^67,68^

The energy landscapes are visualized using disconnectivity graphs,^74,75^ which represent local minima and the energy barriers between them and illustrate the organization of the landscape.^1,76^ Disconnectivity graphs simplify the multidimensional character of the energy landscape and provide insight into how molecular properties are encoded.^39^

We identified clusters of energy minima by applying specific energy thresholds, ensuring a comparable number of structures for analysis across all systems. The lowest-energy structures within these clusters were further examined and are referenced throughout the text as selected minima. These minima were reconstructed into all-atom structures using the Protein Chain Reconstruction Algorithm (PULCHRA),^77^ designed to convert coarse-grained protein models into all-atom representations. PULCHRA reconstructs the backbone atoms based on geometric principles that ensure realistic bond angles and dihedral distributions, while side-chain atoms are added using a rotamer library optimized for accuracy and efficiency. The algorithm also applies energy-based adjustments to minimize steric clashes, ensuring that the final structures are physically plausible and suitable for downstream computational analyses.

Additionally, all reconstructed minima, represented by an AMBER potential, were solvated in a TIP3P octahedral periodic box with a layer of water molecules of 6 Å from the border of the periodic box to the solute and neutralized with counterions (3 Na^+^ ions as each system had a net charge of −3). Energy minimization was carried out in two steps: first we performed 0.5 × 10^3^ steepest descent cycles and 10^3^ conjugate gradient cycles with harmonic force restraints of 100 kcal/(mol Å^2^) on the solute atoms, and then we used 3 × 10^3^ steepest–descent cycles and 3 × 10^3^ conjugate gradient cycles without restraints. Afterward, the system was simulated at 300 K for 10 ps with harmonic force restraints of 100 kcal/(mol Å^2^) on solute atoms and equilibrated for 100 ps at 300 K and 10^5^ Pa in the isothermal isobaric ensemble (NPT). This minimization step was performed using the AMBER ff14SB force field.^78^

For the reconstructed structures, we performed conformational analysis, taking into account properties such as the Root Mean Squared Deviation (RMSD) from a reference structure, Root Mean Square Fluctuations (RMSF), the radius of gyration, solvent-accessible surface area (SASA), and secondary structure properties as well as the contact maps. RMSD calculations were performed using Biopython^79^ after the alignment of all structures. RMSF values were calculated using Biopython by aligning each structure to a reference structure based on main chain atoms and carbon atoms in side chains and then computing the positional deviations across all aligned structures for each residue. Radii of gyration were calculated using the “rgyr” cpptraj module from the AMBER package. Distances for contact maps were determined with the application of the “distance” cpptraj module. For each analyzed group of structures corresponding to minima derived from the energy landscape, we compute the mean and standard deviations of these distances. SASAs were calculated using the “FreeSASA” library.^80^ To define secondary structure elements for residues we employed the Define Secondary Structure of Proteins (DSSP) algorithm within the “mkdssp” software.^81^ The structures corresponding to selected minima were visualized using the UCSF ChimeraX software.^82^

### II.C First Passage Time Analysis

In order to analyze the dynamics of the energy landscape, we must first compute the transfer rates between minima directly connected by a transition state. In the harmonic approximation to transition state theory the transfer rate from state *j* to state *i* is given by,^83−87^
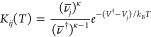
3where the subscripts correspond to minima indexes and † refers to a transition state; *V* is the potential energy; ν̅ is the geometric mean of the normal-mode frequencies; and κ is the number of vibrational degrees of freedom. *k*_B_ is Boltzmann’s constant and *T* is temperature. We do not use the normal-mode frequencies within the UNRES potential because it is a coarse-grained formulation. Vibrational analysis is possible within this framework,^88,89^ but for the estimate in the present survey we simply use ν̅_*j*_ = ν̅^†^ = ν_av_, for all minima and transition states, where ν_av_ is an average frequency factor. It is then convenient to consider the time in units of 1/ν_av_.

The master equation describing the time evolution of the system under the assumption of Markovian dynamics between the directly connected local minima is^90−92^
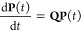
4where **P**(*t*) is the time-dependent vector of occupation probabilities for the local minima. The transition matrix is **Q** = **K** – **D**, where **K** is the rate matrix containing elements *K*_*ij*_ and **D** is a diagonal matrix of escape rates, with elements *D*_*jj*_ = ∑_γ_*K*_*γj*_.

We focus on the computation of first passage times (FPT), which are defined as the time taken to first reach the sink state (i.e., the product), from a given initial starting state (i.e., the reactant). As we are interested only in the first hitting time, no probability can escape the sink, and the corresponding escape rates are set to zero. We can then work within the reduced state space , where , Ω is the full state space, and  is the state space of the sink.  is the subset of the full transition matrix **Q** containing the interstate transition rates within .  is the corresponding subset of **D** including the escape rates to . The master equation describing the modified dynamics becomes
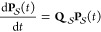
5where  is the occupation probability vector for all minima within .

To compute FPT distributions, we first decompose the transition matrix into its constituent eigenmodes,
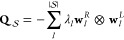
6where  and  are the left and right eigenvectors and ⊗ is the outer product.  is a row vector, and  is a column vector. All eigenvalues are real and negative, . Using the above decomposition and the master equation, we can also write the FPT distribution as a sum over eigenmodes,
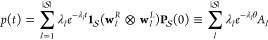
7which defines the amplitude of eigenmode , , a product of dot products. Here,  is a row vector of ones and  is the initial occupation probability in , at *t* = 0. As rates are exponentially sensitive to energy barriers, we make the transformation *y* = ln *t*, to produce the probability distribution ,
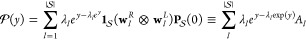
8As *p*(*t*) and  are normalized distributions, .

Each individual eigenmode makes a peak contribution of  to the FPT distribution, which has a peak height of  at . However, as many amplitudes are small in magnitude, there are normally only a few dominant modes that significantly contribute to the FPT distribution. Additionally, we use the right eigenvector components of each eigenmode to determine the relative contribution of each state to the time scale of each individual eigenmode peak. This approach enables us to assign peaks in FPT distributions to sets of minima in the landscape.^93^

In practice, performing eigendecomposition of  can be challenging due to computational limits and especially numerical precision. For the energy landscapes considered in this manuscript, many FPT distributions can be computed using standard eigendecomposition procedures within LAPACK for the full transition matrix  at *T* = 300 K. However, this method breaks down for some transitions, particularly for slow transitions between minima at the bottom of different funnels that constitute kinetic traps. The eigenvalues corresponding to the long time peaks are then many orders of magnitude smaller than other eigenmodes within the system, which results in a separation of time scales and loss of precision in eigendecomposition. This problem causes long time behavior to be computed incorrectly. To overcome this limitation, we use partial Graph Transformation (pGT),^93,94^ a network reduction procedure that removes states from the system, while preserving FPT distributions. This reduction procedure can improve the condition number of the network and enable the accurate computation of eigenmodes corresponding to the slow, rate-determining transitions, which are often the most interesting events. Therefore, to compute FPTs between minima at the bottom of different funnels that are separated by large energy barriers, we use pGT to retain only the states at the bottom of competing funnels before performing eigendecomposition. The pGT procedure also facilitates the computation of FPTs for larger landscapes.

### II.D Hydration Free Energy

Hydration free energy (HFE) is an important thermodynamic property that quantifies the energy change associated with a gas phase solute being dissolved in water.^95,96^ It depends on the interactions between the solute molecules and water molecules and is calculated as the Gibbs free energy difference between the solvated state of a molecule and the gas phase.
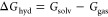
9In a broader sense, the HFE demonstrates how favorable it is for a molecule to be surrounded by water compared to being in a vacuum, which correlates directly to its solubility and stability in aqueous solutions. This property is important for predicting how molecules behave in biological systems where water is the predominant solvent.

For our Aβ28, Aβ40, and Aβ42 databases we performed computations of Δ*G*_hyd_ at the classical GFN-FF level^97^ and with the semiempirical quantum mechanical (SQM) method GFN2-xTB.^98^ Input structures were selected from the Aβ28, Aβ40, and Aβ42 energy landscapes. For simplicity and computational efficiency, all of the investigated structures were optimized at the faster GFN-FF level and rotational–vibrational free energy contributions were obtained thereafter in the modified rigid-rotor harmonic-oscillator (mRRHO) approximation.^99,100^ This procedure has proved to perform reasonably well for macromolecules.^101,102^ The calculations were repeated for both the gas phase and with implicit solvation, modeling solvation free energy effects with the analytical linearized Poisson–Boltzmann (ALPB) model,^103^ parametrized for water. GFN2-xTB calculations with and without ALPB implicit solvation were performed as single-point evaluations of the GFN-FF optimized structures. All calculations were performed with version 3.0 of the CREST code.^104,105^ Since more than one structure for each system was investigated, the final free energies were Boltzmann-weighted to obtain *G*_solv_ and *G*_gas_, which is standard practice for supramolecular calculations.^106^ Subtracting these Gibbs free energies for the two phases finally provides an estimate of Δ*G*_hyd_ according to eq 9, which corresponds directly to solubility.

### II.E Solubility Prediction Using Graph Convolutional Networks

The GCN is a type of neural network designed to process graph-structured data. With each layer of a GCN, the hidden representation of each node is updated according to the hidden representation of its neighbors. The precise propagation protocol is written as^107^
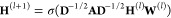
10

Here, **H**^*l*^ represents the hidden representation matrix at layer *l*. The hidden representation is updated with each layer of the GCN. At layer 0, the hidden representation of each node is equivalent to the input node feature vector. **A** is an adjacency matrix, or edge matrix, of the graph with self-connections; it weights the importance of edges for propagating representations across neighbors. **D** is a diagonal matrix used to normalize the adjacency matrix so that each row sums to 1, *D*_*ii*_ = ∑_*j*_*A*_*ij*_. **W**^(*l*)^ is a trainable weight matrix specific to layer *l. σ* represents a nonlinear activation function, in our case the rectified linear unit function, ReLU(*x*) = max(0, *x*).

To convert a protein structure to a graph representation, each amino acid is represented by one node. In the case of a binary edge matrix, an edge between node *i* and *j* is defined (*A*_*ij*_ = 1) if the residues they represent are determined to be in contact, with *A*_*ij*_ = 0 otherwise. Following the Critical Assessment of protein Structure Prediction (CASP) definition, contact means that the inter-residue distance between the C^β^ (or C^α^ for Glycine) is 8 Å or less.^108^

The GCN used here is a previously published model with pretrained parameters called *GraphSol*.^109^ For the solubility prediction, *GraphSol* reports an accuracy of *R*^2^ = 0.48. For context, other machine learning (ML) methods achieve lower accuracy on the *eSol* data set^110^ (*R*^2^ = 0.44 for *SOLart*,^111^*R*^2^ = 0.45 for *ProGAN*(112)). *GraphSol* is a framework to predict the solubility of proteins using only the sequence. Reliance on sequence data is common for ML methods because the database of protein sequences vastly outnumbers the database of experimentally characterized protein structures. A graph representation is constructed, with the edge values being the probability of contact between residues as predicted by SPOT-contact, an ML method to predict contact maps from sequence.^113^ In addition to two GCN layers, *GraphSol* adopts a self-attention layer to pool the variably sized hidden state matrix into a fixed-size protein representation, followed by a sigmoidal multilayer perceptron to map this representation to an output ∈ (0, 1).

The node feature matrix contains 94 features for each node, or , with *N* number of nodes/amino acid residues. The first 20 dimensions of each node feature vector, *H*_*i*_^0^ for node *i*, is derived from the BLOck SUbstitution Matrix BLOSUM62.^114^ This is a 20 × 20 matrix that gives a score for the similarity between each amino acid pair. This encoding has been shown to outperform simple one-hot encoding,^115^ which would imply that each amino acid is strictly orthogonal. The next 50 dimensions refer to 20 features from the Position-Specific Scoring Matrix (PSSM)^116^ and 30 features from the Hidden Markov Matrix (HMM).^117^ These are methods of encoding evolutionary conservation in residues with multiple sequence alignment performed on existing databases, Universal Protein resource (UniProt) Reference clusters UniRef90^118^ and Uniclust30,^119^ respectively. The first 20 dimensions of the PSSM and HMM represent the probability of an amino acid existing at a given position in evolutionarily similar proteins, while the remaining 10 dimensions of the HMM represent aggregate probabilities of insertions or deletions. Additionally, seven physiochemical properties of each amino acid^120^ and predicted structural features from Structural Property prediction with Integrated DEep neuRal network 3 (SPIDER3)^121^ (14 dimensions) were included. SPIDER3 is trained to predict secondary structure and solvent-accessible surface area from sequence-only information, but we can also obtain this information directly from molecular coordinates after our coarse-grained structures were mapped to all-atom representations.

For amyloid solubility prediction, the three features that encode the probability of secondary structure were replaced as one-shot encodings; the presence of a coil, sheet, or helix was calculated with the DSSP algorithm.^81^ The feature that represented the solvent-accessible surface area was also replaced by the calculation from DSSP. Furthermore, the edge feature matrix was replaced by a binary contact map, as there is no longer a probability associated with contact once a structure is defined. These feature replacements were made only during amyloid solubility prediction, and not during training, as the training data set only contains sequence information.

The training set for *GraphSol* was the solubility database of ensemble *Escherichia coli* (*E. coli*) proteins (*eSOL*).^110^ The solubility, *s*, of proteins in the *eSOL* database was measured as the ratio of the supernatant fraction to the total fraction after centrifugation in a cell-free translation system that only contains the essential *E. coli* factors responsible for protein synthesis,^122^ resulting in a value between 0 and 1. This solubility measurement is different from the physical solubility, defined as the concentration of a protein in a saturated solution. The physical solubility of proteins is heavily influenced by pH, salt concentration, etc., and requires higher experimental complexity to measure; thus, it is difficult to construct large data sets with physical solubility.

Because *GraphSol* utilizes a self-awareness layer, we can investigate the self-awareness weights to understand how much each node contributes to the final solubility prediction. This structure enables us to estimate the relative contribution of each amino acid to solubility. *GraphSol* employs two GCN layers; because of these layers, a node’s hidden feature vector has been affected by its neighbors up to two steps away. A self-attention layer with four attention heads then pools these hidden node features into a single hidden feature vector, to which a final convolution is applied to output a single solubility score. We examined the relative contribution of each node to the end solubility prediction by applying attention layer weights to their corresponding hidden node vectors, followed by the final convolution. We did not apply the sigmoidal function during the final convolution, as it is nonadditive and monotonic. Instead, we analyzed the linear outputs before the sigmoid activation, which preserves the relative contributions of each node to the predicted solubility.

## III Results and Discussion

### III.A Hydration Free Energies

The hydration free energies were calculated for selected minima from the Aβ energy landscapes and then Boltzmann-weighted. The corresponding values are presented in Table 1. In general, more negative Δ*G*_hyd_ values indicate a better solubility of the investigated system. When calculating these values using implicit solvation models, it is essential to account for size effects introduced by the implicit solvation potential. Specifically, the solvent-accessible surface area is proportional to the nonpolar surface interaction energy and is directly related to the overall system size. This consideration is particularly important for Aβ monomers, as their size correlates with an increasing proportion of hydrophobic residues: 11 hydrophobic amino acids in Aβ28 (39%), 23 hydrophobic amino acids in Aβ40 (58%), and 25 hydrophobic residues in Aβ42 (60%). Therefore, for comparison, we normalized the overall values by the number of residues to address the impact of increasing the system size and the influence of the hydrophobic surface energy on the analysis. In the context of Aβ monomers, this normalization is crucial, given that hydrophobicity increases significantly with the number of residues, particularly as residues 29–42 are known to be hydrophobic.

**Table 1:** Hydration Energies and Hydration Free Energies, Calculated for the Selected Minima from Aβ28, Aβ40, and Aβ42 Energy Landscapes at the GFN-FF Level of Theory and Boltzmann-Weighted^a^

		Δ*E*_hyd_			Δ*G*_hyd_		
database	method	[*E*_h_]	[kcal mol^–1^]	[kcal mol^–1^]	[*E*_h_]	[kcal mol^–1^]	[kcal mol^–1^]
**Aβ28**	GFN-FF	–0.3851056	–241.66	–8.63	–0.3992255	–250.52	–8.95
	GFN2-xTB	–0.8219843	–515.80	–18.42	–0.8549519	–536.49	–19.16
**Aβ40**	GFN-FF	–0.3867420	–242.68	–6.07	–0.4044057	–253.77	–6.34
	GFN2-xTB	–0.8687382	–545.14	–13.63	–0.9117551	–572.14	–14.30
**Aβ42**	GFN-FF	–0.4083824	–256.26	–6.10	–0.4323021	–271.27	–6.46
	GFN2-xTB	–0.9814659	–615.88	–14.66	–0.9790356	–614.35	–14.63

a Solvent effects were modeled by the ALPB(water) implicit solvation potential. (*) Additionally, values have been divided per number of residues (N) for each system.

Analysis at the GFN-FF level reveals a clear trend in both the hydration energy (Δ*E*_hyd_), excluding rovibrational free energy contributions, and the HFE. According to the force field predictions, Aβ28 is expected to be the least soluble, while Aβ40 shows slightly better solubility, and Aβ42 demonstrates the highest solubility among the three. However, when the results are considered normalized by the number of residues, a different trend emerges. Specifically, Aβ28 appears to be the most soluble, whereas Aβ40 and Aβ42 exhibit very similar solubilities, however significantly lower than Aβ28. This observation supports the well-established concept regarding solubility for Aβ monomers, i.e., that the solubility decreases with the system size. This result underscores the importance of normalizing values to account for size effects. Interestingly, HFE values at the GFN2-xTB level exceed those of GFN-FF by more than a factor of 2, which hints at an underestimation of the intramolecular interactions by the force field. A probable cause for this issue is the description of electrostatics, which become important, especially in combination with the polar energy contributions of implicit solvation and which are typically much better described at the GFN2-xTB level compared to GFN-FF.^98^ Nonetheless, the SQM method exhibits the same general trend as results using HFE: post-normalization, it predicts solubilities for Aβ40 and Aβ42 far lower than that of Aβ28.

### III.B The UNRES Aβ28 Energy Landscape

**Figure 4:**
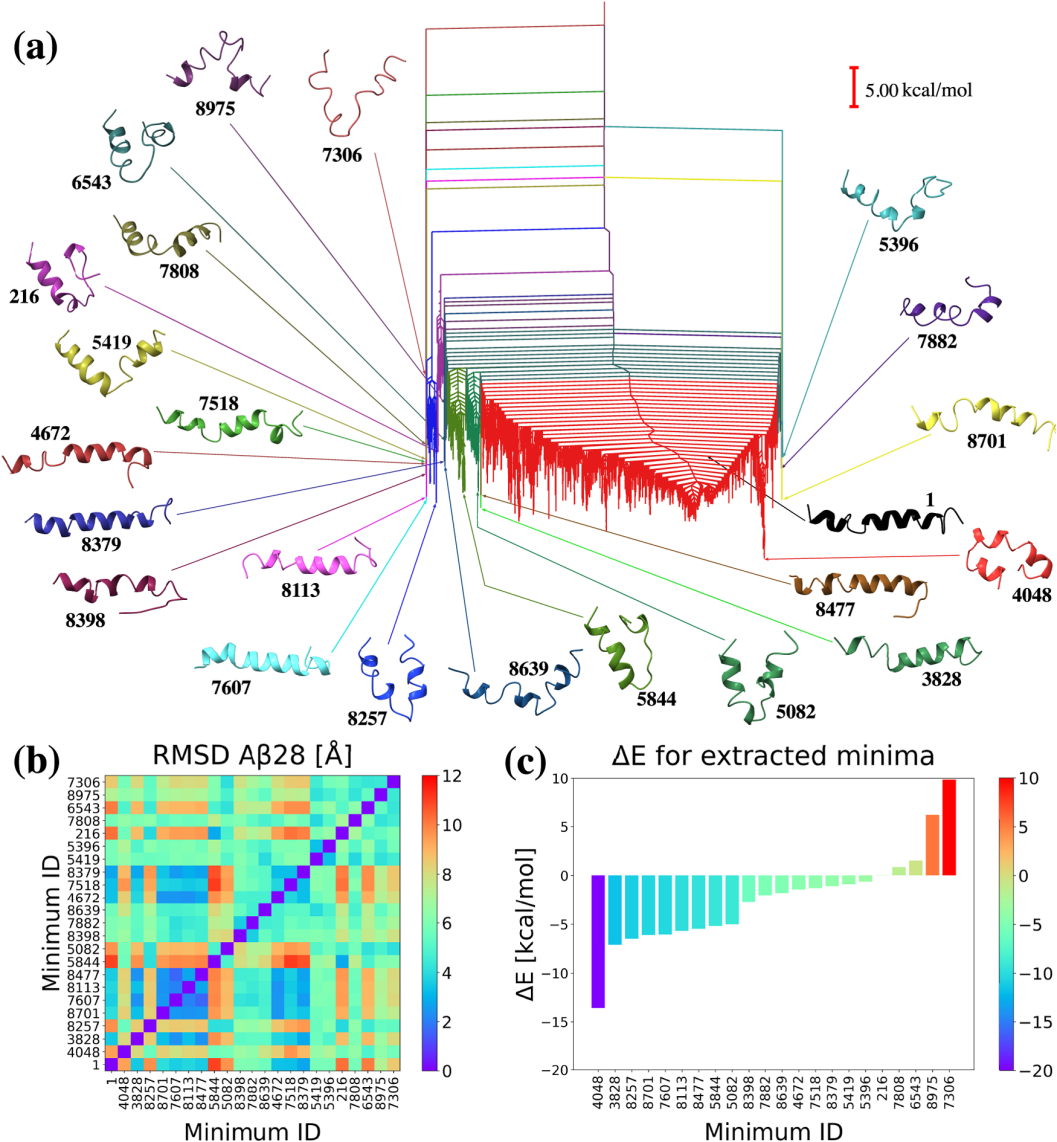
(a) Disconnectivity graph for the Aβ28 UNRES energy landscape, with 22 distinctive groups of minima in different colors. The selected minima are shown and are colored to match their respective clusters and labeled with numbers that refer to the order from the database. Minimum of 1 is the PDB structure, and the global minimum is 4048. (b) RMSD values for the 22 minima after alignment with the PDB structure. (c) Δ*E* for the 22 minima relative to the PDB structure.

First, we performed potential energy landscape explorations for Aβ28 by conducting single-ended searches. This process generated a kinetic transition network with 9072 minima and 11121 transition states. We defined a threshold energy of 14 kcal/mol to cluster the minima into 22 different funnels within the energy landscape, as mentioned in the introduction. These clusters, alongside the selected minima, are shown in different colors in Figure 4a. We calculated UNRES potential energies for the lowest minimum within each cluster relative to the PDB structure, as shown in Figure 4c. Additionally, we calculated the RMSD matrices (Figure 4b) for all of the structures with the lowest energy within each cluster. Finally, Figure 4a illustrates the disconnectivity graph^74,75^ for the Aβ28 UNRES potential energy landscape, with the selected minima and their positions indicated by arrows. Structures are assigned numerical identifiers corresponding to the order in which they were found in the database, with the PDB structure labeled as 1.

Our investigation identified several minima with distinct structures, frequently characterized by more collapsed conformations compared to the straight helical PDB structure. For example, the global minimum of 4048, which is 13.608 kcal/mol lower in energy than the PDB structure (Figure 4c), comprises two parallel segments. The first segment includes the Ala2-Ser8 helical fragment, followed by a turn in the Ser8-Glu11 region. The second segment contains the Glu11-Val24 helix, which is interrupted by a hydrophobic fragment from Leu17 to Phe19, with the characteristic Phe19 residue exposed to the solvent (Figure 4a).

The structure that differs the most from minimum 1 is a minimum of 5844 (Figure 4b), with an RMSD of 10.99 Å after alignment with the PDB structure. This structure begins with the Ala2-Glu15 helical fragment, followed by a sharp turn in the Gln15-Leu17 region. The remaining residues form a coil, with the characteristic Phe19 residue exposed to the solvent, and a short Asp23-Val24 turn. In Figure 4b, there is a visible cluster of four minima (8701, 7607, 8113, and 8477) that are similar in terms of RMSD, each with an RMSD lower than 3 Å after alignment with the PDB structure. The main structural difference within this set of minima is the length of the helix, which is longest in minimum 7607, extending from Ala2 to Val24. Most minima in this set exhibit a break in the helix at Val24. The only minimum where the helix breaks earlier is 8701, which features a Val12-Glu22 helix. Out of these four minima, minimum 8701 differs the most in structure, as it also includes a random coil in the His6-Val12 segment and a short helix from Ala2 to His6.

Some of the selected minima, such as 8257, 5844, and 5082, exhibit significant structural changes and have energies lower than those of the PDB structure (Figure 4a). The length of the random coil separating helices with sharp turns varies among these minima. In minimum 8257, the Ser8-Val18 sequence of turns shifts the structure away from a straight helix, with Val24 exposure to the solvent breaking the helix. In minimum 5844, the Gln15-Lys28 fragment lacks a well-defined secondary structure, featuring turns caused by the hydrophobic Val24 collapsing into the center of the structure. This minimum retains a helical segment from Ala2 to Gln15. Minimum 5082 exhibits a central turn at His14-Gln15, shifting the structure into a V-shape, with two helical fragments, Ala2-Gly9 and Ala21-Val24. Similar to the other minima, the exposed Val24 breaks the helix at the C-terminal end. All three minima seem to exhibit turns associated with Gln15.

Interestingly, a low Δ*E* does not necessarily correlate with a high RMSD relative to the PDB coordinates. This behavior is consistent with the properties of amyloid peptides, which are characterized by multifunnel energy landscapes. Previous studies^28,123^ have demonstrated that these landscapes lack a well-defined global minimum. Instead, they feature many competing structures in the low-energy regions with distinct secondary structures. Some of these structures may resemble or differ significantly from the PDB structure, resulting in arrangements that appear somewhat random and do not directly correlate with a high RMSD relative to the PDB coordinates.

**Figure 5:**
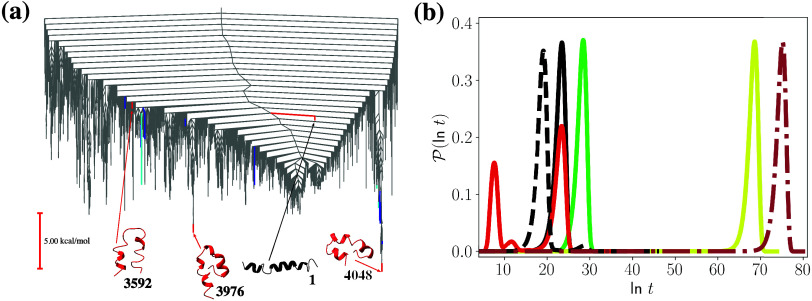
(a) Disconnectivity graph showing the 7525 minima in the red funnel illustrated in Figure 4, for the Aβ28 energy landscape. Minima shown in dark and light blue correspond to the states that contribute most to the time scale of the fastest and the second fastest peaks, respectively, for the FPT distribution for the 4048 ← 3592 transition, which is shown in red in (b). (b) First passage time distributions for transitions within the Aβ28 landscape at *T* = 300 K, 4048 ← 1 (solid black), 4048 ← 3828 (solid green), 4048 ← 8701 (solid yellow), 4048 ← 3592 (solid red), 3976 ← 1 (dashed black) and 7306 ← 1 (dot-dash reddy-brown).

To highlight distinct dynamical time scales and probe competing kinetic pathways, we compute FPT distributions between selected minima (Figure 5). First passage time distributions for transitions between minima at the bottom of funnels in the energy landscape are single-peaked, as the time scales for these transitions are dominated by the time taken to leave the initial trapping basin. The larger the energy barrier between the source and sink, the slower the transition. The slowest transition from the PDB structure is to a minimum of 7306, which is the only minimum with no well-defined secondary structure. Each individual peak in an FPT distribution arises due to a set of pathways between the source and sink that occur on similar time scales. The FPT distribution for the transition from the PDB structure to the global minimum, i.e., 4048 ← 1, is also a single peak. Pathways for this transition generally spend time in state 3976 en route to sink state 4048.

However, multiple peaks can be seen in the FPT distributions for certain starting states. For example, the FPT distribution for the 4048 ← 3592 transition exhibits three peaks. The peak at longer times corresponds to pathways that visit the small trapping basin containing state 3976. The two faster peaks come from pathways that do not enter the 3976 kinetic trap. The states that contribute most to the time scale of the first two peaks are shown in blue in Figure 5.

### III.C The UNRES Aβ40 Energy Landscape

**Figure 6:**
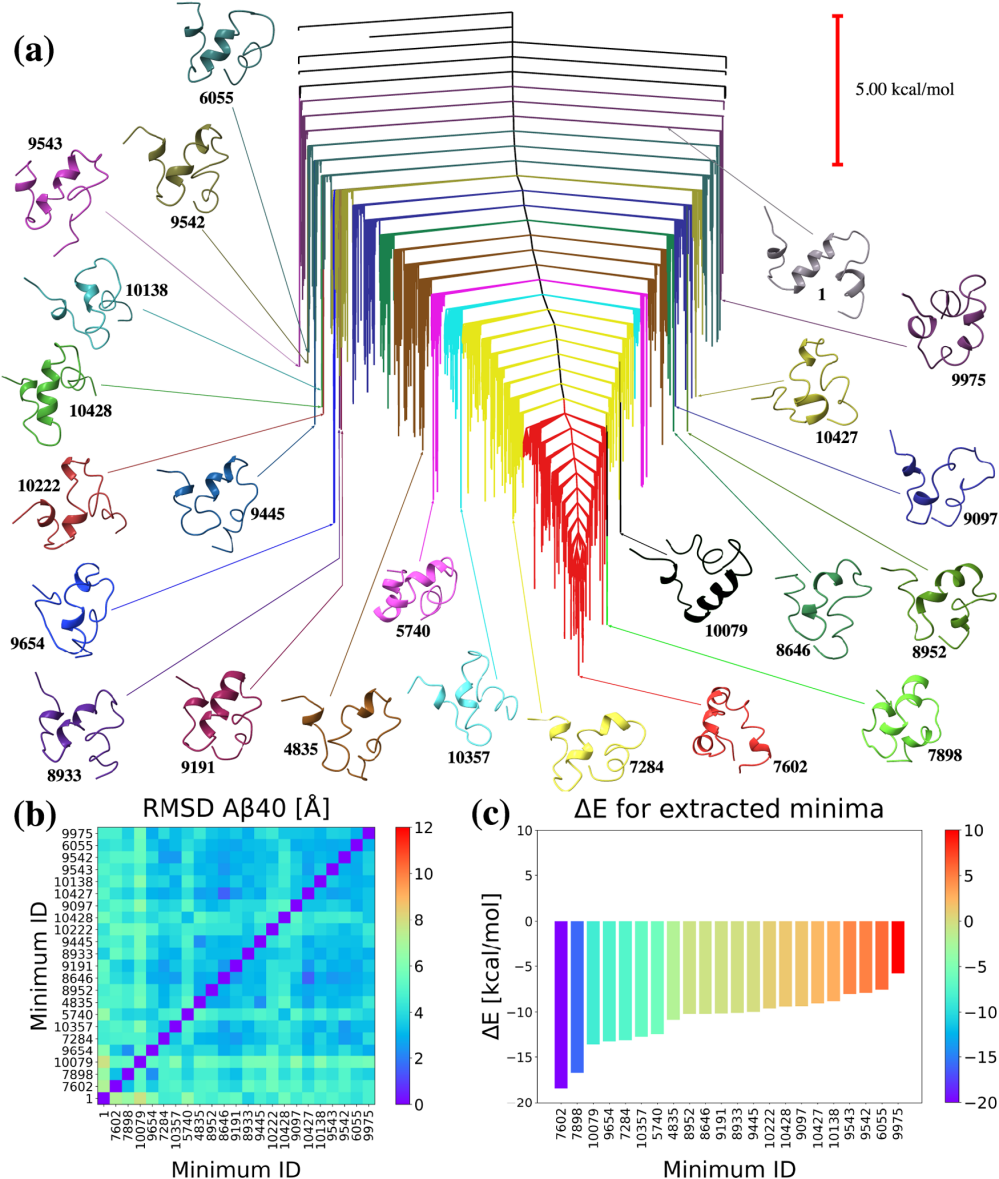
(a) Disconnectivity graph for the Aβ40 UNRES energy landscape, showing 21 distinctive groups of minima in different colors. The selected minima are shown and are colored to match their respective clusters and labeled with numbers that refer to the order from the database. The minimum of 10079, which does not belong to any of these groups, is also included for further analysis. Minimum 1 is the PDB structure, and the global minimum is 7062. (b) RMSD values for the 22 minima after alignment with the PDB structure. (c) Δ*E* for the 22 minima relative to the PDB structure.

The Aβ40 monomer contains 12 additional residues compared to Aβ28, all of which are hydrophobic. Using the same methodology, we first conducted single-ended searches of the potential energy landscape of Aβ40, constructing a kinetic transition network with 11451 minima and 13834 transition states. We defined a threshold energy of 5 kcal/mol and identified 21 clusters of minima based on this criterion. The energy landscape of Aβ40 differs significantly from that of Aβ28, exhibiting a single-funnel topology that leads to a well-defined global minimum. Consequently, the identified clusters of minima represent distinct subsets within the same funnel (Figure 6a). The selected energy threshold was set to 5 kcal/mol to ensure a comparable number of structures for structural analysis. Some minima, such as 10079, do not reside within a trapping basin and are indicated in black. We, therefore, conducted a detailed analysis of a minimum of 10079 and added it to selected minima for Aβ40 (Figure 6).

Our investigation revealed that many minima have energies lower than those of the PDB structure; interestingly, the PDB structure is significantly higher on the Aβ40 landscape than on the Aβ28 landscape. Furthermore, the RMSD matrices (Figure 6b) indicate much smaller structural differences compared with the structures analyzed for the Aβ28 landscape. Despite this trend, several minima exhibited distinctive structures, often more collapsed than the PDB structure. For example, the global minimum, 7602, with an energy 18.440 kcal/mol lower than the PDB structure (Figure 6c), is much more compact with three short helices: Phe4-Gly9, Hie14-Ala21 with Phe19 exposed to the solvent, and Ile31-Gly37. The structure contains two turns significantly influencing its shape: Glu11-Hie13 and Asn27-Lys28. The rest of the global minimum is mostly random coil.

The structure that differs the most from the PDB structure is a minimum of 10079, which is not within the trapping basin. This structure exhibits a very short helical Phe4-Asp7 fragment and a longer Val12-Asp23 helical fragment. The hydrophobic C-terminal end is mostly disordered but partially shifts into contact with the main helix; specifically, Leu17 and Ala30 are intriguingly close in this structure. Phe19 is within the helix and is exposed to the surface. Examining Figure 6b, we find that structures 9445, 8933, 9191, 8646, 8952, and 4835 form one set with smaller RMSD values, below 3 Å, while structures 9097, 10427, 10138, 9543, 9542, and 6055 form another set. Most structures within these clusters exhibit a short helical Phe4-Asp7 fragment. Phe19 is within the helix in most structures; however, the length of this helix varies among different minima, ranging from Hie14-Val24 to shorter Val18-Val24 fragments. Other regions of the structures are rather disordered and lack a well-defined secondary structure.

**Figure 7:**
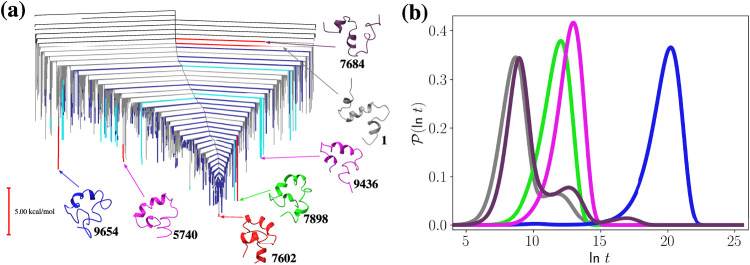
(a) Disconnectivity graph highlighting specific minima within the Aβ40 energy landscape from Figure 6. Minima shown in dark and light blue correspond to the states that contribute the most to the time scale of the fastest and the slowest peaks, respectively, for the FPT distribution for the 7602 ← 1 transition, which is shown in gray in (b). (b) First passage time distributions for transitions within the Aβ40 landscape at *T* = 300 K, 7602 ← 1 (gray), 7602 ← 7898 (green), 7602 ← 9654 (blue), 7602 ← 5740 (pink) and 7602 ← 7684 (purple).

As for Aβ28, we compute FPT distributions between selected minima (Figure 7). As the Aβ40 landscape is single-funneled, there is a smaller range of transition time scales within the network, compared to Aβ28. Once again, transitions between the lowest minima of trapping basins give single-peaked FPT distributions. Transitions from high energy minima to low energy minima can result in multipeaked FPT distributions, such as those shown for the 7602 ← 1 and 7602 ← 7684 transitions. The states that contribute the most to the time scale of the fastest and slowest peaks for the 7602 ← 1 transition are shown in dark and light blue, respectively, in the disconnectivity graph in Figure 7. Unlike Aβ28, the time scales of these peaks are due to contributions from many different minima, rather than only a few, as the UNRES landscape has a single funnel. Despite the landscape exhibiting a single funnel, some minima have larger barriers to escape the funnel than others, e.g., the minima labeled in light blue in Figure 7a. Pathways that visit these light blue minima during the 7602 ← 1 transition contribute to the slower peak in the FPT distribution, as it takes longer to escape from these minima. Because some minima have slower escape times, multipeaked FPT distributions appear for many different transitions between high-energy and low-energy states.

### III.D The UNRES Aβ42 Energy Landscape

**Figure 8:**
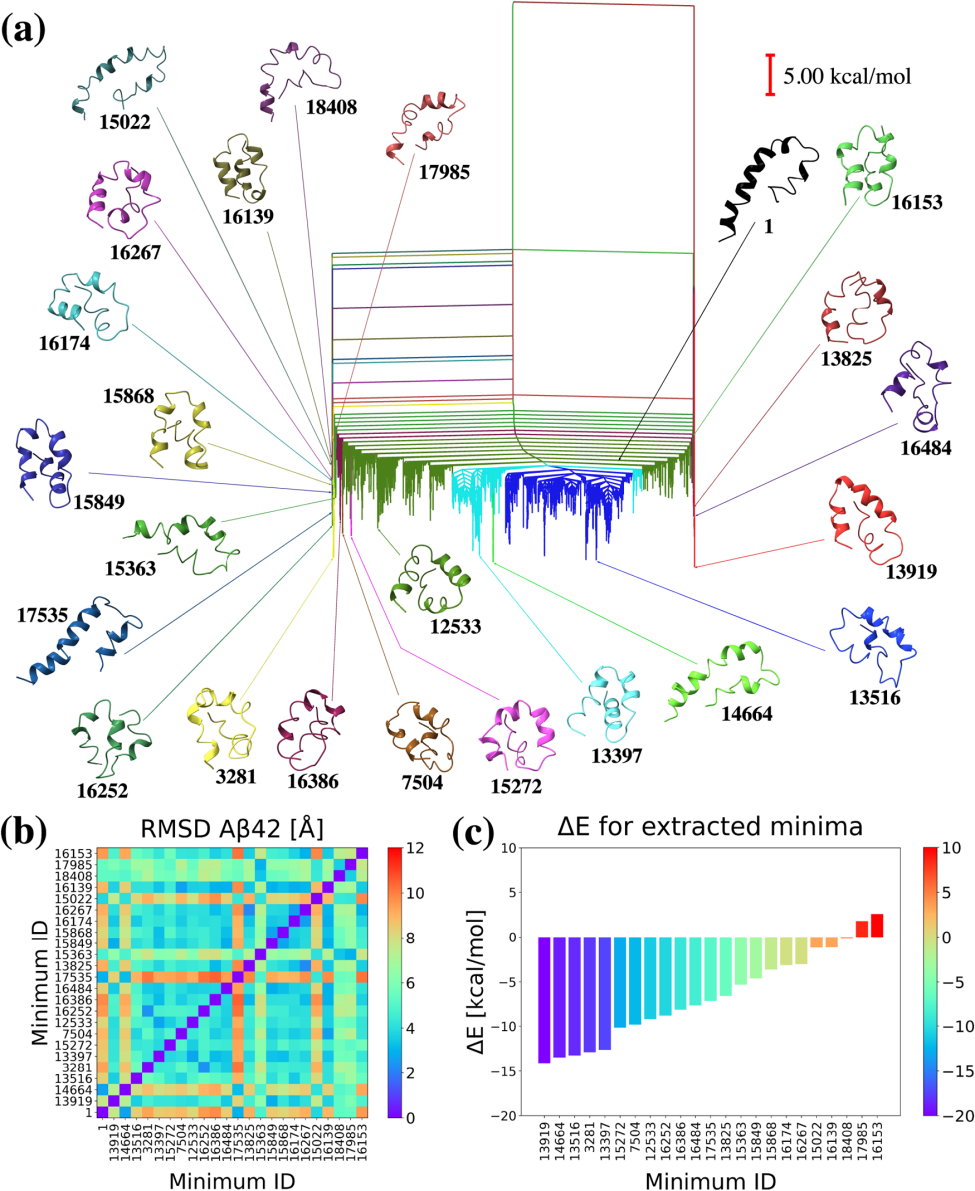
(a) Disconnectivity graph for the Aβ42 UNRES energy landscape, with 23 distinctive groups of minima in different colors. The selected minima are shown and are colored to match their respective groups and labeled with numbers that refer to the order in the database. Minimum 1 is the PDB structure, and the global minimum is 13919. (b) RMSD values for the 23 minima after alignment with the PDB structure. (c) Δ*E* for the 23 minima relative to the PDB structure.

Aβ42 has two additional hydrophobic residues Ile41 and Ala42 compared to Aβ40. We employed the same methodology and first conducted single-ended searches of the potential energy landscape of Aβ42, establishing a kinetic transition network comprising 18429 minima and 24919 transition states. We then defined a threshold energy of 11 kcal/mol and grouped the minima into 23 disjointed trapping sets based on this criterion. We performed the same analysis as before for structures with the smallest energy of each set (Figure 8). Interestingly, the multifunnel energy landscape of Aβ42 differs significantly from that of Aβ40 and the organization is like the UNRES results for Aβ28. Most of the selected minima lack secondary structure and have high potential energy barriers between them, as observed for different atomistic potentials in previous studies.^28,124^

The global minimum, 13919, which has an energy similar to that of the next lowest energy minimum, is separated by the highest transition state (Figure 8a), highlighting the substantial energy increase required to reach other competing minima with similar energies (Figure 8c). This result demonstrates the importance of thorough exploration of the complex amyloid energy landscape (Figure 8a), as minima with similar energies can differ significantly in structure when separated by substantial barriers.

The RMSD matrix (Figure 8b) shows that the Aβ42 structures differ much more significantly than those in the Aβ40 energy landscape. For instance, all selected minima except 17535 and 14664 differ significantly from the PDB structure, with only three minima maintaining the straight helix fragment at the N-terminal end, albeit with different fragment lengths: Ala2-Ser26 for minimum 1, Ala2-Val24 for minimum 17535, and Glu3-Val24 for minimum 14664. These minima exhibit the greatest divergence from other structures, which often break this helix in the hydrophobic C-terminal end, attempting to collapse inside the structure and leaving the N-terminal end without a well-defined straight helix, as observed in the mentioned structures. Additionally, other clusters with moderate RMSD values below 4 Å are present (Figure 8b), including minima 16267, 16174, 15868, 15849, and 16139. In most of these structures, Phe19 is within the helix and consistently exposed to the solvent, which may further influence the breaking of the helix within the Phe20-Val24 region. This region, rich in hydrophobic residues, appears to cause further structural changes and shifts the rest of the hydrophobic C-terminal end into the center of the structure in most cases (Figure 8a). The Aβ42 network is ill-conditioned and more complex than the other landscapes, making FPT analysis challenging. However, we plan to conduct this analysis in future work.

### III.E Further Structural Analysis

**Figure 9:**
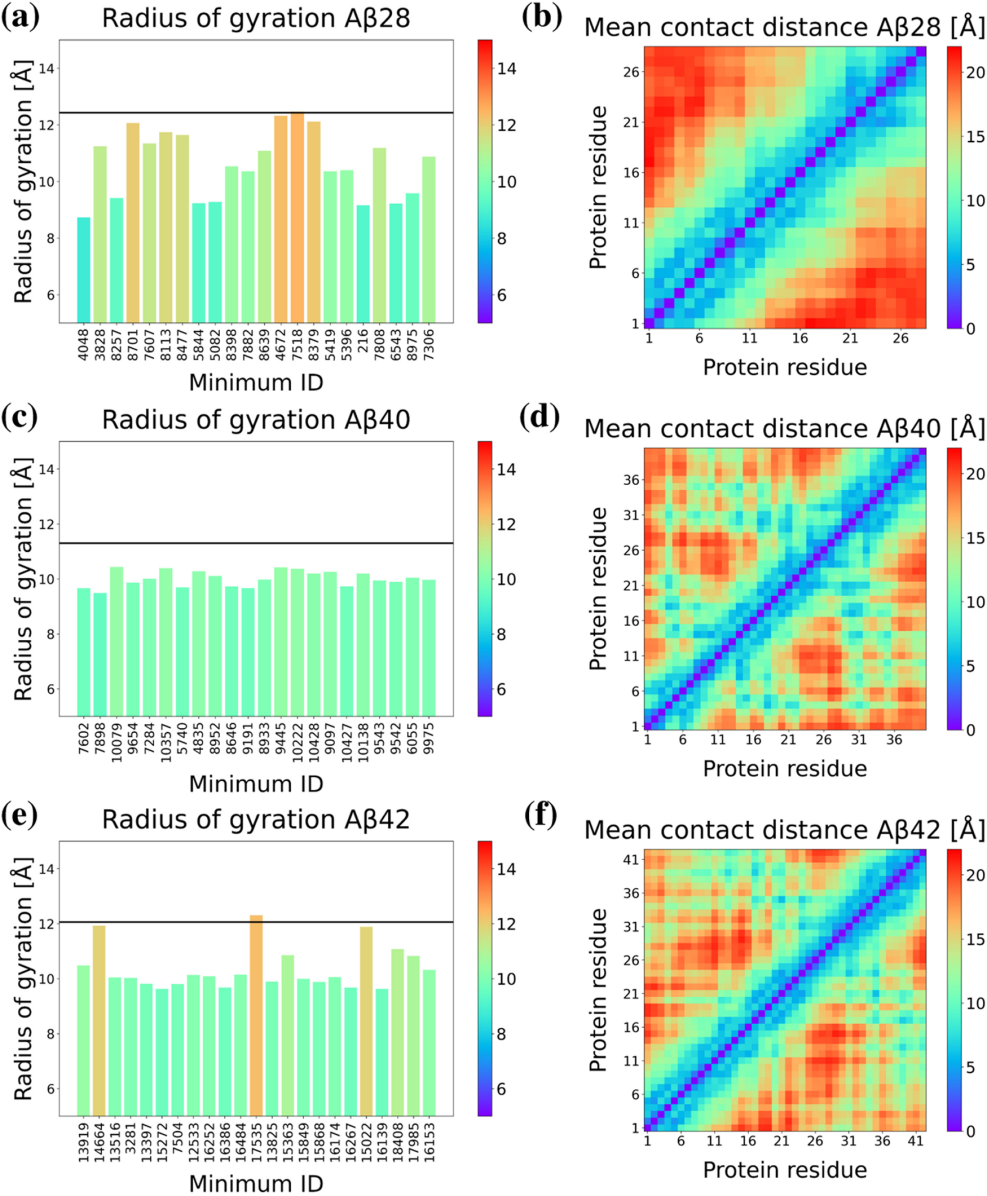
(a, c, e) Radius of gyration and (b, d, f) mean contact maps for selected minima from Aβ28, Aβ40, and Aβ42 databases, respectively. Mean contact maps were averaged over all selected minima from each database.

We examined selected minima in terms of the radius of gyration (Figure 9a,c,e) to identify trends and assess the compactness of the structures. The results are largely consistent with the RMSD matrices. In each case, the radius of gyration for the minima is lower than for the initial PDB structure. This observation aligns with expectations for the experimentally determined PDB structures (1AMC, 1AML), which were obtained using solid-state NMR. Solvation during simulation induces further disruption of the straight helical fragment observed in the experimental structure. For 1IYT, hexafluoroisopropanol (HFIP) was used, during solution NMR, in structure determination. HFIP is known to stabilize helices, similar to trifluoroethanol; therefore, helix disruption was anticipated when our computations were performed without HFIP.^125^ For Aβ28, structures with the highest radius of gyration also showed the least deviation from the initial PDB structure (Figure 9a). Notably, the radius of gyration varies significantly across the minima studied in the Aβ28 energy landscape. In contrast, the results for the Aβ40 minima are much more consistent (Figure 9c), displaying only minor changes in the radius of gyration. This observation suggests that most of these structures are more compact, which was also evident during the structural analysis of the minima. It is consistent with the fact that the first minimum obtained by minimizing a PDB structure lies relatively high on the energy landscape, indicating that more compact structures with less exposed hydrophobic regions are energetically favorable according to UNRES. Similarly, structures identified on the Aβ42 landscape with a straight helix also have a significantly higher radius of gyration (Figure 9e).

Additionally, we constructed a map of contacts (Figure 9b,d,f) averaged over all extracted minima for each system. The contact map for Aβ28 (Figure 9b) reveals a moderately dispersed pattern of contacts, indicating that the peptide adopts structures that are not uniformly compact. Several off-diagonal contacts suggest interactions between residues that are not sequentially close, pointing to the presence of secondary structural elements such as loops or turns. There are regions with denser contacts, which likely correspond to stable secondary structures, such as α helices. This dispersed contact pattern is consistent with the higher and more variable radii of gyration values observed for Aβ28 (Figure 9a), suggesting less compact and more structurally diverse conformations. The contact map for Aβ40 (Figure 9d) exhibits a more uniform and denser pattern of contacts compared to that of Aβ28. This result indicates that Aβ40 adopts more compact and stable structures with UNRES. Several contiguous blocks of contacts are evident, indicating regions where residues are in close proximity and likely form stable secondary structures. Diagonal regions with high contact density suggest the presence of α-helices, while off-diagonal blocks indicate turns. This uniformity and density of contacts correlate with the more consistent and lower radius of gyration values (Figure 9c), indicating that the structures are more compact. The contact map for Aβ42 (Figure 9f) displays a mix of dense and sparse regions, reflecting both compact and more extended structural elements. Regions with high contact density, similar to Aβ40, suggest the presence of stable secondary structures. Additionally, there are more dispersed contact regions, corresponding to the straight helical structures, which contribute to higher radii of gyration values. This variability in contact density suggests a combination of compact structures and extended helical elements. The mixed contact pattern aligns with the higher and more variable radius of gyration values observed for Aβ42 (Figure 9e), reflecting the structural diversity of Aβ42 with both stable folded regions and extended helices.

We further investigated the significance of hydrophobic and polar regions given that these regions are known to be the primary structural differences among Aβ monomer structures. This analysis is particularly crucial for Aβ monomers, as their size correlates with an increasing proportion of hydrophobic residues: 11 hydrophobic amino acids in Aβ28 (39%), 23 hydrophobic amino acids in Aβ40 (58%), and 25 hydrophobic residues in Aβ42 (60%). Table 2 reflects this trend, showing that with increasing monomer size, there is a rise in total SASA, and notably, a significant increase in hydrophobic contribution from 30.22% for Aβ28 to 47.04% for Aβ42. For Aβ42, the hydrophobic and polar SASA contributions are nearly equal, which significantly affects the structure, with a significant number of hydrophobic residues exposed to the solvent.

**Table 2:** Averaged SASA for Selected Minima from the Aβ Databases

database	total [Å^2^]	polar [Å^2^]	hydrophobic [Å^2^]	polar [%]	hydrophobic [%]
**Aβ28**	2908.69 ± 143.28	2029.01 ± 113.63	879.68 ± 94.63	69.78	30.22
**Aβ40**	3370.32 ± 124.09	1806.19 ± 99.70	1564.13 ± 94.93	53.59	46.41
**Aβ42**	3483.86 ± 130.67	1843.88 ± 108.10	1639.98 ± 141.98	52.96	47.04

**Figure 10:**
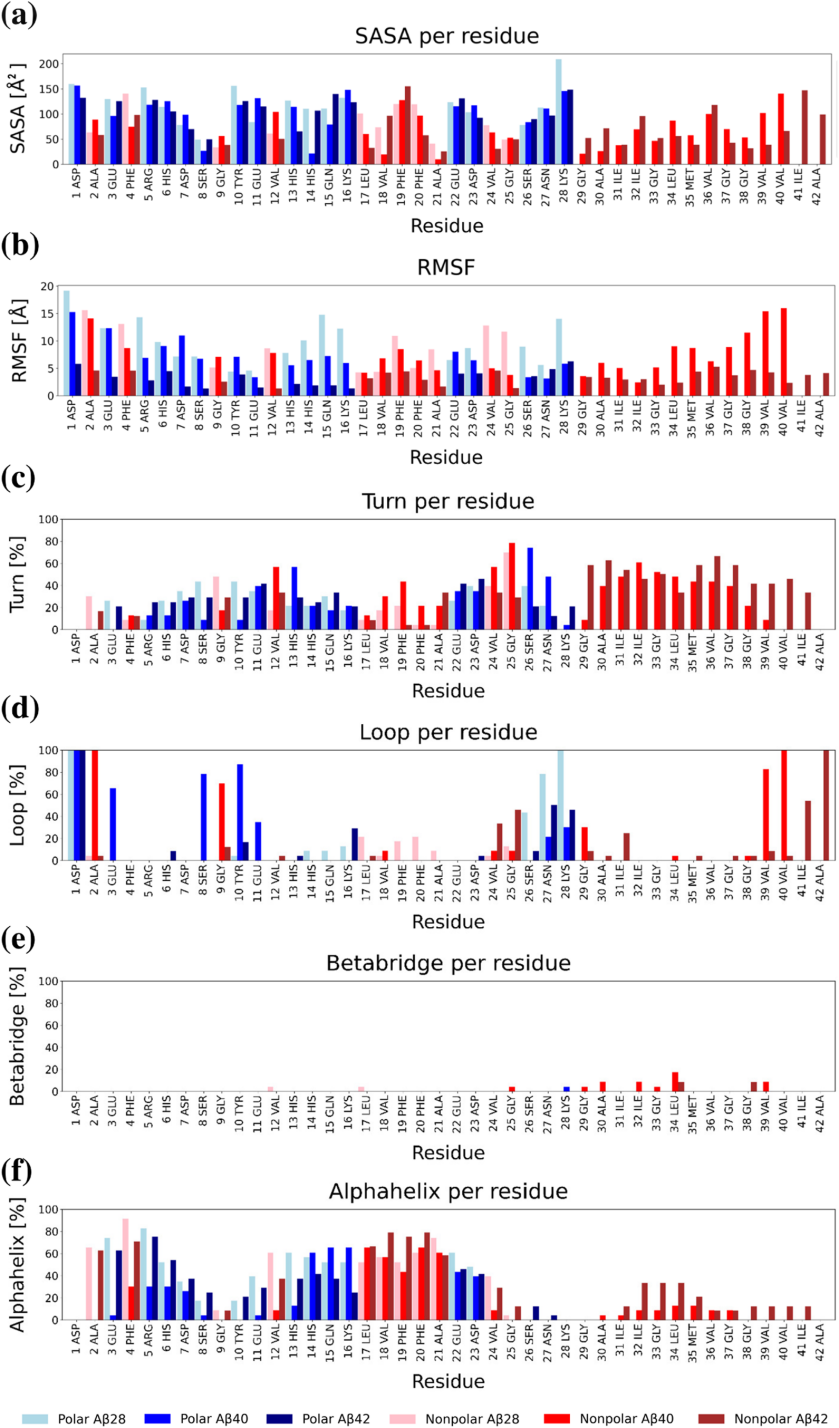
(a) SASA per residue. (b) RMSF per residue. (c–f) Secondary structure contribution per residue calculated with the DSSP algorithm. All data have been calculated after averaging results for all of the selected minima. The figure contains a legend with different color codes for polar and hydrophobic residues for different databases.

Next, we investigated the contribution of specific residues to the SASA across different databases. Figure 10a shows the average SASA per residue for the various databases. The trends vary with the sequence length, with residues at the termini generally exhibiting the highest SASA. Hydrophobic residues adjacent to polar ones tend to be buried, resulting in a decreased SASA. This trend is particularly evident in the increasing SASA for the hydrophobic C-terminal end of Aβ40 and Aβ42. An interesting observation arises for hydrophobic Phe19, which consistently has the highest SASA among all hydrophobic residues, increasing significantly with the size of the Aβ monomer. This residue is often solvent-exposed in the selected minima. Additionally, it is apparent that with increasing system size, the contribution of polar residues to the SASA decreases. The lowest SASA values were consistently observed for Ser8, Gly9, and Ala21 across all systems.

In Figure 10b, we analyzed the RMSF of residues, calculated as the positional deviations across all aligned structures for each residue within the given system. Notably, the residues with the highest fluctuations were consistently associated with the termini. Interestingly, the fluctuation also appears to correlate with system size, as residues in Aβ42 exhibited the lowest RMSF compared to Aβ28 and Aβ40. Specifically, at the C-terminal end, the small structural diversity of the hydrophobic region is more pronounced in Aβ42 compared to Aβ40.

We also predicted the secondary structure distribution using the DSSP algorithm (Figure 10c–f). This analysis allowed us to identify residues in different systems, based on averaged structures, that are particularly prone to forming helices, turns, β-bridges, and random loops. Interestingly, β-bridges are not evident upon visual inspection of the structures, yet the algorithm defined some structures as particularly prone to developing these interactions. This discrepancy could be attributed to various factors, such as the influence of the UNRES potential on secondary structure development or undersampling of parts of the energy landscape where β structures would be present. It is also important to realize that visualization programs may define and display secondary structures differently. The assignments are therefore interpreted as suggestions.

For Aβ28, Val12 and Leu17 appear prone to β-interaction at one minimum (8975). For Aβ40, several residues were identified in this category: Gly25, Lys28 for 8646; Gly29, Ile33, Gly33, Leu34, and Val39 for 8933; Ala30, Leu34 for 9543; Ala30, Leu34 for 10222; Ile32 for 5740; and Leu34, Val39 for 9975. In Aβ42, two residues, Leu34 and Gly38, were identified as being prone to form β sheets in the monomer form for minima 15272 and 18404. However, the participation of these residues in β-bridge formation is minor, contributing less than 10% of the β-bridges across all minima in the databases and only for the particular structures mentioned above. Most of these residues participate in turn in random coil formation (Figure 10c,d). The terminal residues are typically assigned as loops by DSSP (Figure 10d), and much of the C-terminal hydrophobic region is characterized by a sequence of turns (Figure 10c). The largest contribution of α-helices is visible in Figure 10f, where the His14-Val24 fragment consistently contributes to the α-helix formation. The length of this helical fragment varies, being Val12-Val24 on average for Aβ28. Generally, the helix in this region shortens with an increasing system size. Interestingly, the helix breaks at Val24 where a turn (Gly25-Ser26) occurs and the Asn27-Lys28 region primarily forms loops. The Glu3-Asp7 fragment predominantly forms α-helices, although other types of helical structures, especially 3_10_-helix, are also detected for Phe4-His6 fragment in the Aβ40 databases.

### III.F Solubility

**Figure 11:**
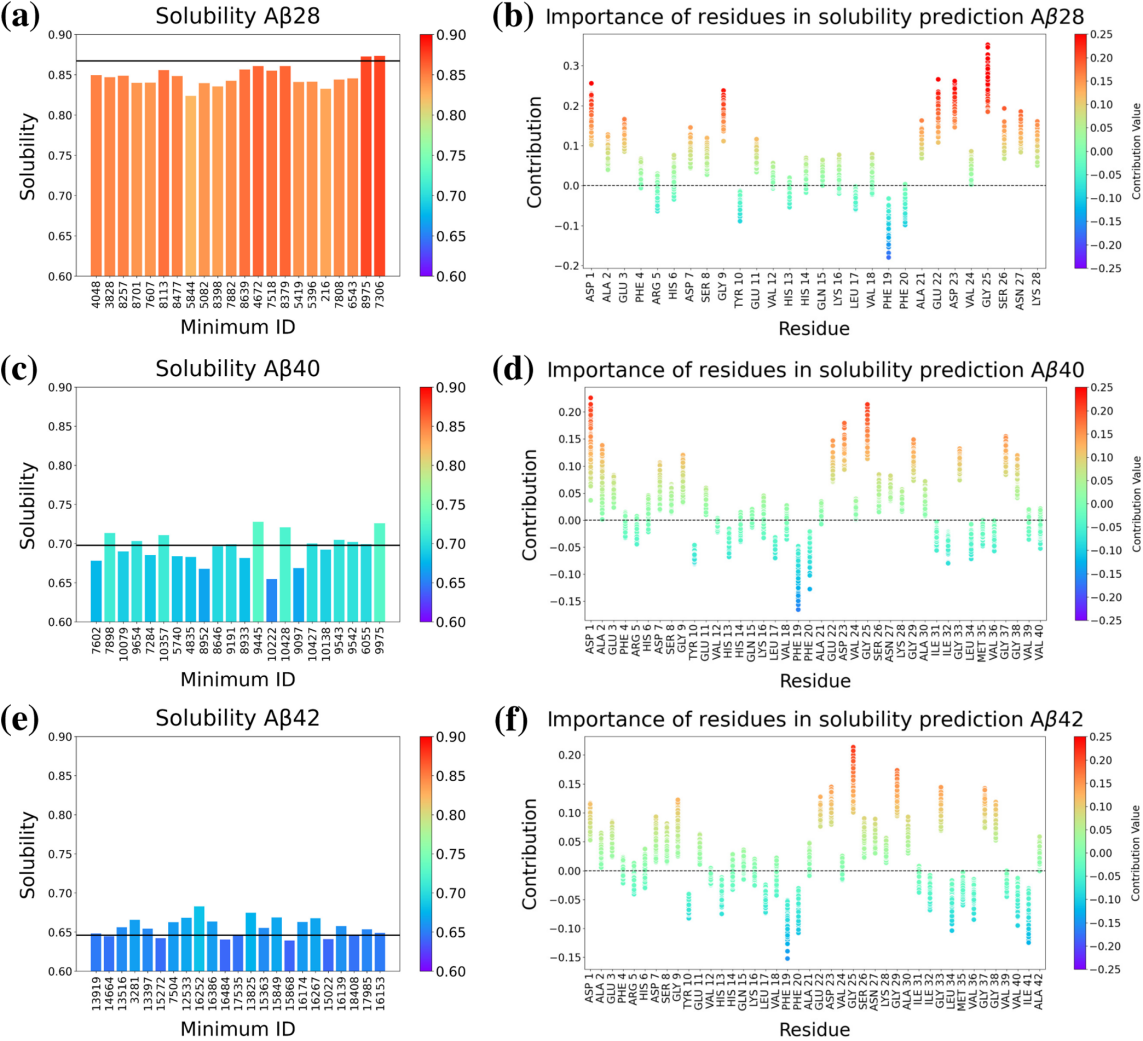
(a, c, e) Solubility predicted by GraphSol for selected minima of Aβ28, Aβ40, and Aβ42. In each panel, the black lines are the predicted solubilities of the PDB structures. (b, d, f) Importance of residues in solubility prediction.

To provide predictions of estimated solubility, we employed GCN fits to data, as discussed in Section II.E. The results are shown in Figure 11a,c,e. Our findings are consistent with the well-established experience in the literature that solubility decreases with increasing size.^126^ The predicted solubility values for different minima did not differ significantly within each database. However, the Aβ28 minima exhibited lower values (Figure 11a) compared to the predicted solubility of the PDB structure for this molecule, while the predicted solubility for Aβ40 and Aβ42 minima exhibit values both below and above the predicted solubility of the original PDB structure (Figure 11c,e).

To further investigate the contributions of specific residues to solubility predictions, we analyzed their importance within the model (Figure 11b,d,f). Note that the contributions for the hidden state of a given residue include contributions from neighbors up to two edges away, with each edge ranging up to 8 Å in distance. The weights in our analysis represent the relative contributions of each residue to the overall solubility prediction of the peptide. It is important to note that we have not applied the final sigmoidal layer to these weights, which would distort the values due to the nonlinear transformation. The importance weights should be understood as relative contributions to the solubility prediction. They can be interpreted in terms of how much each residue (and its surrounding region) contributes, positively or negatively, to the solubility prediction prior to the final sigmoidal transformation. This approach should highlight the individual impact of residues on solubility, providing insights into which of them are most influential in the peptide solubility profile.

**Figure 12:**
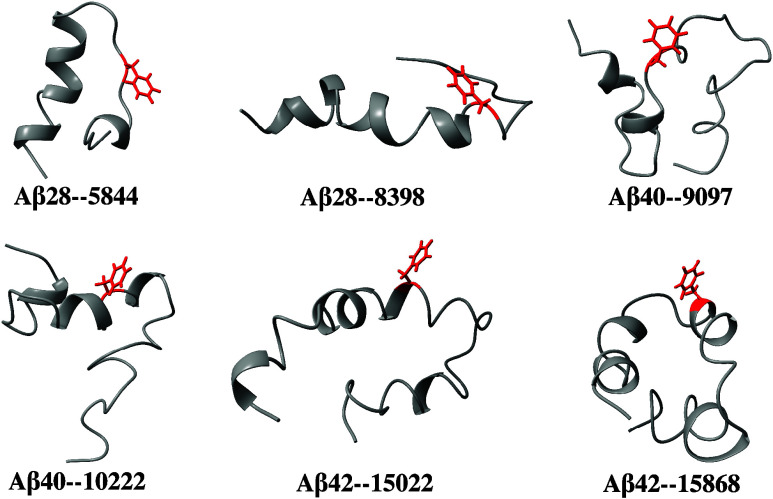
Illustration of various low-solubility minima, with the Phe19 residue highlighted in red.

We can immediately identify the importance of the Phe19 residue and its surrounding region with the most negative contribution to solubility while exhibiting a high variance. This result means that fluctuations in this region play a key role in *GraphSol*’s prediction of overall protein solubility. In Figure 12, we illustrate different low solubility minima with the hydrophobic Phe19 residue exposed to the solvent, resulting in disruption of the helical structure in the surrounding region. Bernstein et al.^127^ experimentally demonstrated the critical role of Phe19 in Aβ aggregation by comparing wild-type Aβ42 to an F19P mutant. While wild-type Aβ42 rapidly formed larger oligomers and aggregates, including hexamers and pairs of hexamers thought to be early steps in protofibril formation, the Pro19 alloform only formed smaller oligomers (dimers, trimers, and tetramers) and did not progress to larger assemblies. For Aβ42, Ile41 also exhibits a substantial negative effect on the solubility.

On the other hand, Gly25 contributed most positively to the predicted solubility across all data sets, with Asp1 also making a notable contribution in the case of Aβ40. Remarkably, the anticorrelation from the C-terminal residues appears to be more pronounced in Aβ42, suggesting a distinct solubility profile influenced by the terminal residues. Previous work indicates that the C-terminal of Aβ monomers is more active in aggregation, fibrillation, and β-strand formation.^128^ This expectation is further corroborated by the minor but still significant β-bridge interaction observed at the C-terminal end (Figure 10e).

All correlations presented here were obtained using Pearson linearization. There is a moderate correlation (*r* = 0.40) between the radius of gyration and solubility for Aβ28, no correlation for Aβ40 (*r* = −0.05), and a moderate anticorrelation for Aβ42 (*r* = −0.49). For Aβ28, less compact structures exhibit a higher solubility, suggesting they would be less prone to aggregation. In the case of Aβ40, the lack of correlation is likely due to the very similar radii of gyration across all of the analyzed structures. For Aβ42, the pattern shifts, with more compact structures featuring a buried hydrophobic core demonstrating higher solubility and, potentially, a reduced tendency to aggregate. Furthermore, there is a low anticorrelation (*r* = −0.29) for Aβ28 between the RMSD after alignment with the PDB structure and solubility. This result seems logical, as the extended PDB structure has a large radius of gyration; thus, structures most similar to the PDB structure will have the highest radius of gyration and the smallest RMSD. For Aβ40, no significant correlation (*r* = 0.16) is observed, likely because the structures do not differ significantly in terms of both radius of gyration and RMSD. However, for Aβ42, there is a moderate correlation (*r* = 0.53), indicating that an increase in RMSD aligns with an increase in solubility. As the PDB structures contain a long α-helix, their radius of gyration is significantly higher. Therefore, more compact structures, identified by a lower radius of gyration, are associated with the highest solubility.

Aβ42 monomers aggregate more readily compared to Aβ40.^129^ The presence of two additional hydrophobic residues in Aβ42 (Ile41 and Ala42) facilitates the formation of inter-residue contacts absent in Aβ40, including hydrophobic clustering between residues Val39-Ile41 and increased clustering among residues Gly37-Gly38 and Val12-Lys16.^129^ These interactions are evident in Figure 10b, where the fluctuations at the C-terminal end of Aβ42 are significantly smaller than those for Aβ40, indicating more stable interactions that predispose Aβ42 to aggregation. Literature indicates that the most rigid structure within both monomers is the hydrophobic cluster around residues Leu17-Ala21.^130^ Rojas et al.^131^ conducted coarse-grained molecular dynamics simulations of five Aβ28 monomers to investigate the dynamics of aggregation. They found that when the distance between residues Leu17 and Ala21 was constrained to be inflexible, β-rich aggregates did not form. This observation is consistent with the results of Kapurniotu et al.,^132^ who demonstrated that the Aβ28 (L17K, A21D) peptide mutant, featuring a helix-stabilizing lactam bridge between Lys17 and Asp21, did not aggregate. Through our structural analysis, we found that a disrupted α-helix surrounding Phe19 produces a predicted solubility. Both contact maps (Figure 9b,d,f) and minimal fluctuations for Leu17 and Ala21, along with a small SASA for these residues, indicate the critical role of stabilization in this hydrophobic region. Disruption, by the exposure of Phe19 to the solvent, results in helix disruption, further emphasizing the importance of this region in maintaining solubility.

Previous studies have identified a key hydrophobic interaction between Phe19 and Leu34 in Aβ40 to produce β-bridges, a feature also observed in our secondary structure contributions per residue (Figure 10e) for Leu34. This contact is consistently reported in structural studies of Aβ fibrils^133^ and has been implicated in oligomer toxicity.^134,135^ Disrupting this interaction significantly reduces membrane affinity for Aβ oligomers,^134^ further highlighting its role in early aggregation. Computational studies also suggest a high probability of intermolecular Phe19–Leu34 contacts in both Aβ40 and Aβ42,^136^ supporting its relevance in β-bridge formation and potential neurotoxicity. Additionally, the observed His14–Val24 propensity for α-helical conformations is consistent with experimental solution structures of micelle-bound Aβ40 and Aβ42, which identify the Gln15–Val24 region as predominantly α-helical.^27^

## IV Conclusions

Our study provides insight into the solubility, structural dynamics, and aggregation tendencies of Aβ28, Aβ40, and Aβ42 peptides using a variety of computational approaches. Hydration free energy (HFE) calculations using the GFN-FF and GFN2-xTB methods revealed subtle solubility trends. Initially, Aβ42 appeared to have the highest solubility; however, after normalizing for size, Aβ28 exhibits the highest solubility, while Aβ40 and Aβ42 exhibit lower solubilities, consistent with the established size-dependent solubility trends in amyloid peptides. The discrepancy between HFE values from the GFN-FF and GFN2-xTB methods highlights possible underestimates of intramolecular interactions by force fields, particularly in implicit solvation models affecting electrostatics and polar energy contributions. This result highlights the importance of methodological considerations in accurately assessing peptide solubility.

Exploration of the energy landscapes of Aβ28, Aβ40, and Aβ42 highlights distinct structural features. Aβ28 and Aβ42 exhibit multifunnel landscapes, associated with disorder.^23,28^ This structural complexity has been associated with the transition from α-helices to β-hairpins, a hallmark of amyloid aggregation and plaque formation.^137^ In contrast, Aβ40 presents a simpler, single-funnel landscape. This result could reflect undersampling of Aβ40 if alternative funnels have been missed, and further investigation is warranted in future work.

Structural analysis involving the radius of gyration and contact maps further highlights the differences between the peptides. Aβ28 displayed variable radius of gyration profiles, suggesting structural diversity and flexibility, whereas Aβ40 appeared consistently compact, indicative of stable configurations. Aβ42 exhibited a mix of compact and extended structures, reflected in its higher and more variable radii of gyration values, correlating with diverse contact patterns indicating both folded and extended helical elements. SASA analysis highlighted increasing hydrophobic residue exposure with peptide size, influencing solubility and aggregation propensity. Hydrophobic Phe19 consistently exhibited high SASA values for all systems, linking its solvent exposure with increased aggregation tendency. Residue-specific analyses identified Phe19, Ile41, and Ala42 as key determinants impacting solubility through their roles in inter-residue interactions and structural stability. Correlations with the radius of gyration and RMSD are consistent with these structural determinants, showing a positive correlation between structural compactness and solubility in Aβ42, where deviations from helical structures increased the aggregation propensity.

Our study employing the GCN method provides further insight into the solubility and structural characteristics of the Aβ28, Aβ40, and Aβ42 peptides, highlighting the aggregation propensity. Consistent with existing literature, Aβ28 is predicted to have the highest solubility, and Aβ42 the lowest. Structural analyses highlight distinct features influencing solubility. Aβ28, despite its smaller size, exhibits configurations with differing radius of gyration, and some structures with higher radius of gyration are predicted to have greater solubility. In contrast, Aβ40 structures, characterized by consistent compactness and minimal structural variance, exhibit solubility profiles that are not strongly correlated with structural changes. For Aβ42, a moderate anticorrelation between solubility and structural compactness was observed, indicating that more compact conformations with buried hydrophobic cores will have a higher solubility and be less prone to aggregate. Key structural determinants impacting solubility were identified through residue-specific analyses. The hydrophobic Phe19 emerges as a key residue, with a significant negative impact on solubility predictions for the three monomers, consistent with experimental results that link Phe19 exposure to solvent with increased aggregation propensity. Regarding dynamics, our study identified a wide range of time scales, with Aβ28 exhibiting the slowest transformations, due to its diverse structural transitions. Furthermore, we observed multipeaked first passage time distributions for relaxation to the global minimum, depending on the initial starting minimum.

Overall, our findings agree with conventional size-based solubility trends in amyloid peptides, providing detailed insights into how structural variations influence solubility and aggregation dynamics. Machine learning methods such as GCN offer predictive capabilities based on structural features, complementing experimental approaches in advancing therapeutic strategies against protein aggregation disorders. Our results highlight the central role of Phe19 in early amyloid assembly, consistent with previous studies identifying Phe19 involvement in hydrophobic interactions critical for aggregation. However, amyloid formation is a multifaceted process, and the aggregation of other intrinsically disordered proteins is driven by additional effects, including electrostatics and sequence-specific interactions. Additionally, emerging evidence suggests that amyloidogenic proteins can form liquid-like condensates, which may serve as intermediates in fibril formation and contribute to proteopathies. Future studies integrating these alternative aggregation pathways could provide a more comprehensive understanding of amyloid self-assembly. Looking ahead, future research will explore the aggregation propensity of selected low-energy structures and investigate the mutational effects on solubility and aggregation. Utilizing the UNRES potential for accelerated simulations, combined with side-chain refinement techniques such as the Pulchra program and relaxation using all-atom potentials like AMBER minimization, provides a valuable balance of precision and efficiency in exploring the global structural landscapes of amyloids. These methods will be further employed in future work.
